# Semi-Automated Computational Identification of Fibrosis for Enhanced Histopathological Decision Support

**DOI:** 10.3390/jimaging12040152

**Published:** 2026-03-31

**Authors:** Alexandru-George Berciu, Diana Rus-Gonciar, Teodora Mocan, Lucia Agoston-Coldea, Carmen Cionca, Eva-Henrietta Dulf

**Affiliations:** 1 Automation Department, Faculty of Automation and Computer Science, Energy Transition Research Center, Technical University of Cluj-Napoca, 400114 Cluj-Napoca, Romania; alexandru.berciu@campus.utcluj.ro; 2 Department of Pathology, Iuliu Hatieganu University of Medicine and Pharmacy, 400012 Cluj-Napoca, Romania; diana.gonciar@umfcluj.ro; 3 2nd Department of Internal Medicine, Emergency County Hospital, 400012 Cluj-Napoca, Romania; luciacoldea@yahoo.com; 4 Department of Physiology, Iuliu Hatieganu University of Medicine and Pharmacy, 400006 Cluj-Napoca, Romania; teodora.mocan@umfcluj.ro (T.M.); carmen.cionca@gmail.com (C.C.); 5 Department of Nanomedicine, Regional Institute of Gastroenterology and Hepatology, 400337 Cluj-Napoca, Romania; 6 PHYSCON Research Center, Óbuda University, H-1034 Budapest, Hungary

**Keywords:** myocardial fibrosis, automation detection, CIELAB color space, Gabor filters

## Abstract

Myocardial fibrosis is a critical prognostic marker involving a progressive cascade of pathological conditions. Accurate assessment of fibrosis in myocardial samples is a routine but difficult procedure for pathologists. This article presents a semi-automated system designed to ease this task while providing pixel-level accuracy that exceeds manual estimation capabilities. The proposed innovative approach combines Gabor filters with CIELAB color space analysis to ensure the efficiency and interpretability of calculations. Testing on histopathological samples, differentiating between fibrous, healthy, and variant tissues, yielded a promising accuracy of 87.5% for images with fibrosis and 80% for all 45 images tested. This system successfully establishes a solid foundation for automated diagnosis, providing pathologists with a reliable and highly accurate tool for quantitative analysis of cardiac tissue.

## 1. Introduction

In 2019, 33% of deaths globally were caused by heart disease [1]. Specifically, out of 55 million deaths, 18.5 million were caused by cardiovascular disease, about 8 million more than the 10.08 million cancer-related deaths. The same trend is found in the United States, where every 33 s a person dies from cardiovascular disease, costing the US healthcare system $239.9 billion between 2018 and 2019 [2]. Myocardial fibrosis is the culmination of a series of cardiovascular diseases [3,4] and involves a validated process of evolution from tissue damage to inflammation to fibrosis [5,6]; therefore, early diagnosis could save millions of lives. Myocardial fibrosis can be identified in multiple cardiac or systemic diseases that clinically evolve with heart failure. Moreover, it distorts the myocardial architecture and changes the composition of the extracellular matrix, therefore being directly involved in the occurrence of heart failure and in its aggravation [7].

Imaging evaluation is essential for establishing the definitive and etiological diagnosis and for providing information with a prognostic role. However, histological analysis provides complementary information about the pattern of fibrosis [8]. Also, microscopic evaluation of the myocardium is an essential part of the autopsy (both clinical and forensic), especially in cases of sudden death [9]. Several aspects represent practical difficulties for pathologists in performing a microscopical evaluation of myocardial fibrosis. The focal nature of the pathological process, the inability to extensively evaluate the heart microscopically, as well as the possible false-negative results following error in sampling in the localized forms of the pathology, require additional tools to be made available for the pathologists to enhance the diagnosis [10]. Recent years have brought a rise in the evolution of multimodal cardiac imaging, especially cardiac magnetic resonance, which allows the in vivo assessment of myocardial composition and even fibrosis through late gadolinium enhancement [11].

At the same time, research on digital tools for enrichment of histological evaluation proficiency, ease of diagnosis, and diagnosis accuracy has gained increasing interest as a solution for histology limitations [12,13]. The use of automated systems for fibrosis detection is an extensively reviewed topic in the literature [14,15,16,17,18,19,20]. To be usable, they need a large set of training images. An example is the Iowa Virtual Slidebox [21]. However, training the networks involves a significant computational effort, which also involves high energy consumption. To counteract these disadvantages, machine learning-based methods are more suitable [22] and are used to delimit regions of interest [23].

To this end, some of the researchers used CIELAB color space to identify fibrosis in the heart [12,24] or kidney [25]. In the CIELAB color space, color is expressed as the three digits L* for perceived lightness and a* and b* for the four different hues of human vision: red, green, blue, and yellow [26]. In the CIELAB, a perceptually uniform space, a given number change should correspond to a corresponding perceived change in color [27]. The LAB space is not perceptually uniform, yet it is useful in industry for detecting subtle color changes [28].

Other research teams have used Gabor filters to process images undergoing convolutional neural network analysis (CNN) [29]. Because of its ideal localization in both the frequency and spatial domains, the Gabor filter, a linear filter, works well as a feature extractor for texture mapping [30]. The team of researchers led by Vukka Snigdha [31] achieved 91% accuracy using the Gabor filters method and the MobileNet deep learning model on a dataset having breast histopathology images with 277,524 patches. Texture analysis is also an important element in the detection of features that the human eye cannot identify and can be performed automatically to detect fibrosis [32,33].

In order to improve the current level of knowledge in the field of using machine learning methods for fibrosis detection, the authors conducted a rigorous review of the literature. To the best of our knowledge, no papers propose the use of a semi-automatic diagnostic system that works for different types of slides and does not use neural networks for the selection of the fibrosis region.

The evaluation of fibrosis is a routine activity for pathologists when evaluating the myocardium. As a result of the above, in order to support pathologists in their repetitive work, the present research proposes a semi-automated diagnostic system to support pathologists. The solution proposed in the paper is based on the analysis of multiple hematoxylin and eosin-stained images in the CIELAB color space and textures using Gabor filters. The research makes two novel contributions to the field. Firstly, it employs CIELAB and Gabor to diagnose myocardial fibrosis, a method intended to withstand varying slide image qualities despite current constraints in testing data. Secondly, it does so without the computational disadvantages of neural networks. In this way, although automation solutions based on CNN [29] or exclusively on the CIELAB color space [24] have been developed and presented in the literature, this paper differs by including Gabor filters, which have proven their superiority in terms of texture analysis, a necessary capability in the field of fibrosis analysis.

## 2. Materials and Methods

**Figure 1:**
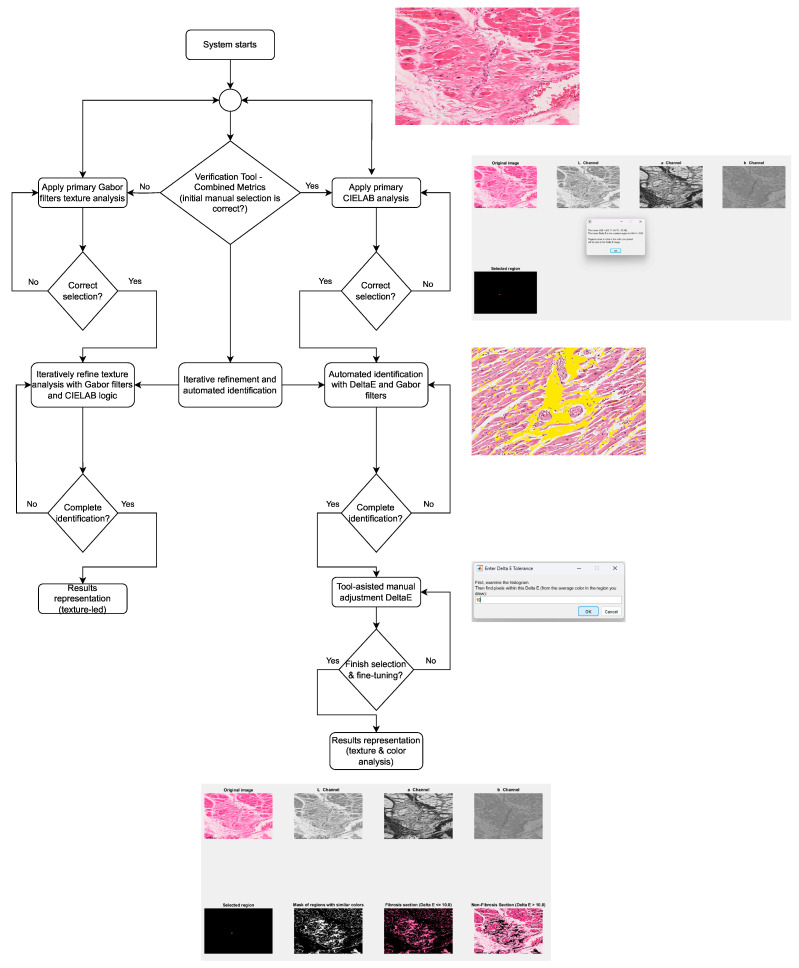
The flowchart of the system.

The CIELAB color space analysis was implemented in both MATLAB^®^ R2024B and Python version 3.12.7, while the testing using Gabor filters was done in MATLAB^®^. For the MATLAB^®^ implementations, version R2024B and the Image Processing Toolbox version 12.1 were used. Regarding the Python implementation, the OpenCV version 4.12.0.88 library and the color-science version 0.4.6 package for Python version 3.12.7 were used. The flowchart of the newly developed system is presented in Figure 1.

The theoretical framework of the mathematical model of the new semi-automated fibrosis diagnosis system is given in Equations (1)–(6). The abbreviations used are as follows:

$$\{\begin{array}{c} V-colour\;variation\;in\;the\;analysed\;image\\ I_{RGB}-original\;RGB\;histopathological\;\;image\\ I_{XYZ}-converted\;image\;to\;XYZ\;colour\;space\\ I_{LAB}-converted\;image\;to\;CIELAB\;colour\;space\\ N-number\;of\;pixels\;of\;I_{RGB}\\ W\;and\;H-width\;and\;height\;of\;I_{RGB}\\ X_{r},\;X_{g},\;X_{b},\;Y_{r},\;Y_{g},\;Y_{b},\;Z_{r},\;Z_{g},\;Z_{b}-\;RGB\;to\;XYZ\;conversion\;coefficients\\ X_{l},\;X_{a},\;X_{b},\;Y_{l},\;Y_{a},\;Y_{b},\;Z_{l},\;Z_{a},\;Z_{b}-\;XYZ\;to\;CIELAB\;conversion\;coefficients\\ I_{G}-converted\;image\;using\;Gabor\;filters\\ C_{time}\;and\;C_{memory}-runtime\;and\;memory\;cost\;functions \end{array}$$

The preprocessing of the histopathological image regarding the color variance is modeled by Equation (1).

(1) $$V\left(I_{RGB}\left(x,\;y,:\right)\right)=\frac{1}{N}\sum_{x=1}^{W}\sum_{y=1}^{H}{\left\|I_{RGB}\left(x,\;y\right)-\mu \right\|}^{2},\;where\;\mu =\frac{1}{N}\sum_{x=1}^{W}\sum_{y=1}^{H}I_{RGB}\left(x,\;y\right)$$

The mathematical model of the conversion to CIELAB color space is given in Equation (2). *XYZ* color space is used as an intermediate to achieve this conversion.

(2) $$I_{XYZ}\left(x,\;y,:\right)=\left[\begin{array}{c} X_{r}\\ Y_{r}\\ Z_{r} \end{array}\;\;\;\begin{array}{c} X_{g}\\ Y_{g}\\ Z_{g} \end{array}\;\;\;\begin{array}{c} X_{b}\\ Y_{b}\\ Z_{b} \end{array}\right]\times I_{RGB}\left(x,\;y,:\right)\;\;I_{LAB}\left(x,\;y,:\right)=\left[\begin{array}{c} X_{l}\\ Y_{l}\\ Z_{l} \end{array}\;\;\;\begin{array}{c} X_{a}\\ Y_{a}\\ Z_{a} \end{array}\;\;\;\begin{array}{c} X_{b}\\ Y_{b}\\ Z_{b} \end{array}\right]\times I_{XYZ}\left(x,\;y,:\right)$$

The application of Gabor filters is based on Equation (3) for feature extraction. The joint color-texture feature vector *F* is defined in Equation (4).

(3) $$\{\begin{array}{c} G(x,y)=[|G_{\theta 1,f1}(x,y){|}^{2},\ldots ,|G_{\theta s,fs}(x,y){|}^{2}]\in R^{s}\\ I_{G}\left(x,\;y,:\right)=I_{RGB}*G\left(x,y\right)\; \end{array}$$

(4) $$F(x,y)=\left[I_{LAB}\left(x,\;y,:\right),\;\;I_{G}\left(x,\;y,:\right)\right]\in R^{3+s}$$

The anisotropic textures related to fibrotic structures are described by the project of the feature vector *F* using a wavelet–Gabor hybrid basis $\phi_{j}^{k}$, where *j* is scale and *k* is orientation (Equation (5)).

(5) $$W_{j,k}\left(x,\;y\right)=\langle F,\;\;\;\;\;\;\phi_{j}^{k}\left(x,\;y\right)\rangle$$

Let the Gabor energy response be E(*x, y*). The superlevel set filtration is included in Equation (6).

(6) $$\Omega_{\alpha }=\{\left(x,\;y\right)\in \Omega \;|\;F(x,y)\geq \alpha \}$$

The graph *G* = (*V*, *W*), computed using k-nearest neighbors over feature vectors *F_i_*. Let *z_i_* ∈ {0, 1} indicate a fibrosis (1) or healthy (0) region. The posterior in Equation (7) is used to model feature distributions.

(7) $$P\left(F_{i}\;|\;z_{i}\right)\propto P\left(z_{i}\;|\;F_{i}\right)\cdot P\left(F_{i}\right)$$

Let ⊝ denote the full set of tunable parameters (Gabor settings, window sizes, classifier hyperparameters). The utility functional is included in Equation (8).

(8) $$L\left(⊝\right)=\lambda_{1}\cdot Dice\left(\hat{z_{i}}\right)-\lambda_{2}\cdot C_{time}\left(⊝\right)-\lambda_{3}\cdot C_{memory}\left(⊝\right)$$

For each implementation *i* ∈ {1, 2, 3} (e.g., MATLAB, Python, Gabor-based), Equation (9) defines the performance vector.

(9) $$S_{i}=\left[{Dice}_{i},\;{Time}_{i},\;{Memory}_{i},\;{TopologyScore}_{i}\right]$$

The final selection is made using a nonlinear scoring function *R* trained via learning-to-rank techniques. Equation (10) defines the final classification step in which the specific candidate region I* that maximizes the nonlinear discriminant function *R* is strictly isolated. This function evaluates each candidate *S_i_* by projecting its composite CIELAB color space and Gabor spatial-frequency textural features into a continuous relevance score, which is calibrated via learning-to-rank optimization to optimally correlate the algorithmic identification of fibrotic tissue with manual ground-truth annotations.

(10) $$I^{*}=argmaxR(S_{i})$$

### 2.1. Protocol Description

Multiple relevant single images were manually extracted with Biolucida Cloud Viewer (2024.2.0) from hematoxylin and eosin-stained whole-slide images available in the histopathology and histology collection of The Iowa Virtual Slidebox [21]. The dataset generated compresses 40 images with myocardial fibrosis with different severity and 5 images where fibrosis was not apparent (2 with normal myocardium and 3 with other lesions). For validation purposes, the collagenous area was manually selected and labeled by a pathologist, being defined as the pale eosinophilic and fibrillar material deposited in the extracellular space.

### 2.2. Fibrosis Detection Using CIELAB Color Space

Taking into account its advantages, the solution tested in the present research was based on the findings of the Image Analyst MATLAB^®^ user [22]. This implementation involves choosing a region of a geometric shape determined by the user. To evaluate the additional accuracy generated by this implementation, the present research implements and tests a solution using Python in which the region selected by the user is always rectangular, with dimensions determined by the user based on mouse positioning.

**Figure 2:**
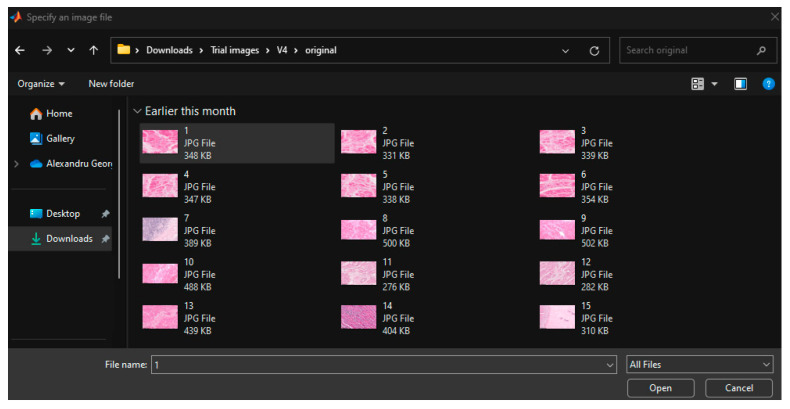

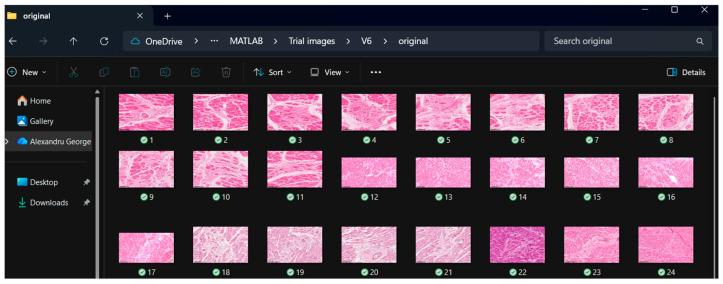
The first step in using the system involves selecting the image from a folder where the original images are stored. Original images: © The Iowa Virtual Slidebox [21].

To begin with, in the case of implementation in MATLAB^®^, the user of the system chooses the histopathological image he wants to submit to the automatic analysis of the system, according to Figure 2.

**Figure 3:**
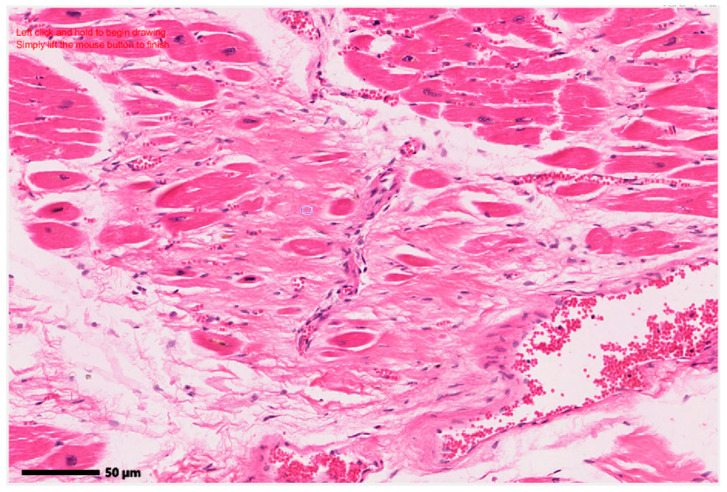
Selection step of the region considered to have fibrous tissue by the pathologist (region of irregular shape), from the original image. Original image: © The Iowa Virtual Slidebox [21].

Given the objective of the program to assist medical staff in accurately identifying myocardial fibrosis from histopathological images, the system requires the user to select a region that they consider to possibly have fibrosis. This selection is made with the mouse, using the original image chosen by the user, and the user can select a region of any geometric shape, as in Figure 3.

**Figure 4:**
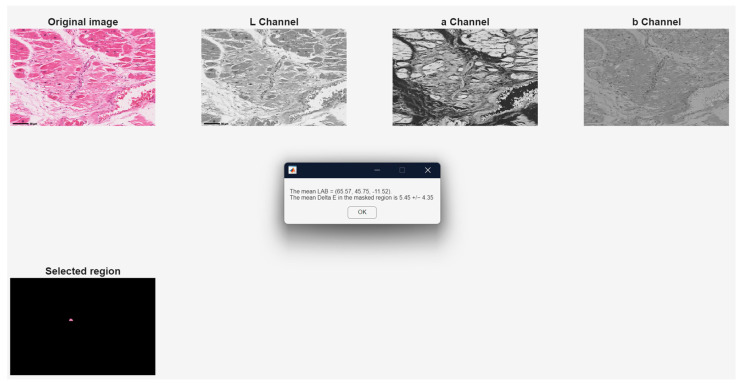
The step of informing the user about the DeltaE variation in the selected region. Original image: © The Iowa Virtual Slidebox [21].

In the next step, the program plots the image in the LAB color space and calculates Delta E, an indicator that represents the color variation in the LAB color space for the region selected by the user, using the mean of the standard deviation for each of the three channels. The results obtained in this step are shown in Figure 4 for one of the analyzed images.

**Figure 5:**
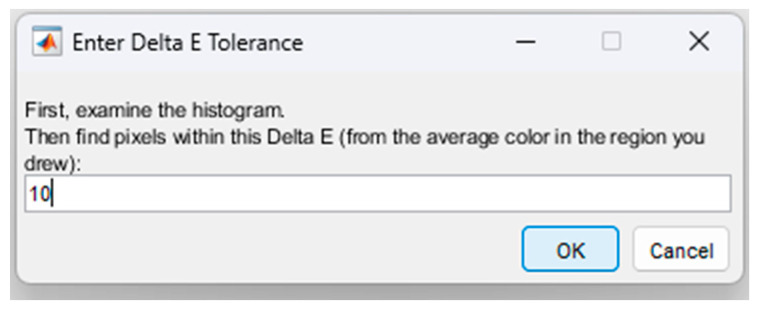
The step by which the standard value for DeltaE tolerance is imposed.

If the user wants to enter another value for the Delta E tolerance, in the next step, the system asks whether to keep the value determined automatically in the previous step or to enter a new value. The higher this value is, the wider the color variation will be, and this will lead to the identification of tissue with fibrosis over a larger area than the real one. Considering the tests carried out in the present research, a value of 10 was imposed for DeltaE tolerance, as shown in Figure 5.

In the last step, the program displays in a graphical format the original image; its representation in L, a, and b channels; the region selected by the user; and the mask of the regions having the same color as the region selected by the user. If it is identical, this means that it falls within the tolerance limits imposed for Delta E = 10 and the color variation in the user-selected region. To identify fibrosis, it was not necessary to divide the set of images into training and testing, as the solution was based exclusively on a mathematical model of pixel- and region-level analysis.

In the end, the system includes in the displayed image the sections of the original image that have the same color as the one selected by the user, identified as having fibrosis, and the regions that have not been selected, being without fibrosis and with different colors from the region selected by the user.

**Figure 6:**
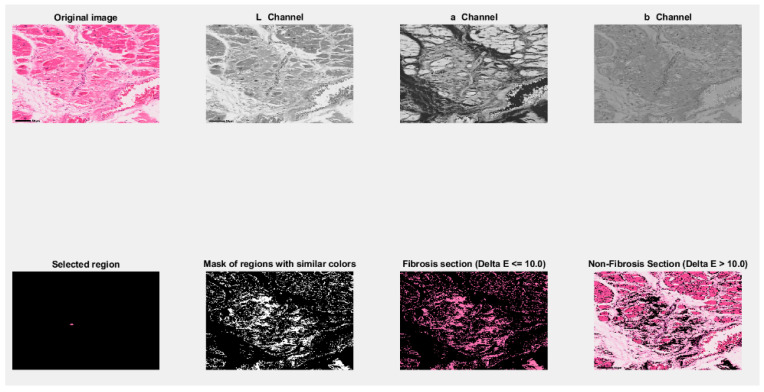
The graphical user interface of the results for MATLAB^®^ implementation of CIELAB color space analysis. Original image: © The Iowa Virtual Slidebox [21].

The graphical interface in which the results are presented to the pathologist is shown in Figure 6. By accurately selecting the region of interest, the system can identify the region with fibrosis. Validation of the results was carried out manually with the help of experts in the field of medical and histopathological image interpretation.

The results obtained were generated by keeping the DeltaE tolerance constant and equal to 10 across the 45 histopathological images analyzed. The value of the variable was determined following a series of tests carried out. The DeltaE tolerance was kept equal for all images analyzed to increase the objectivity of the method accuracy analysis.

**Figure 7:**
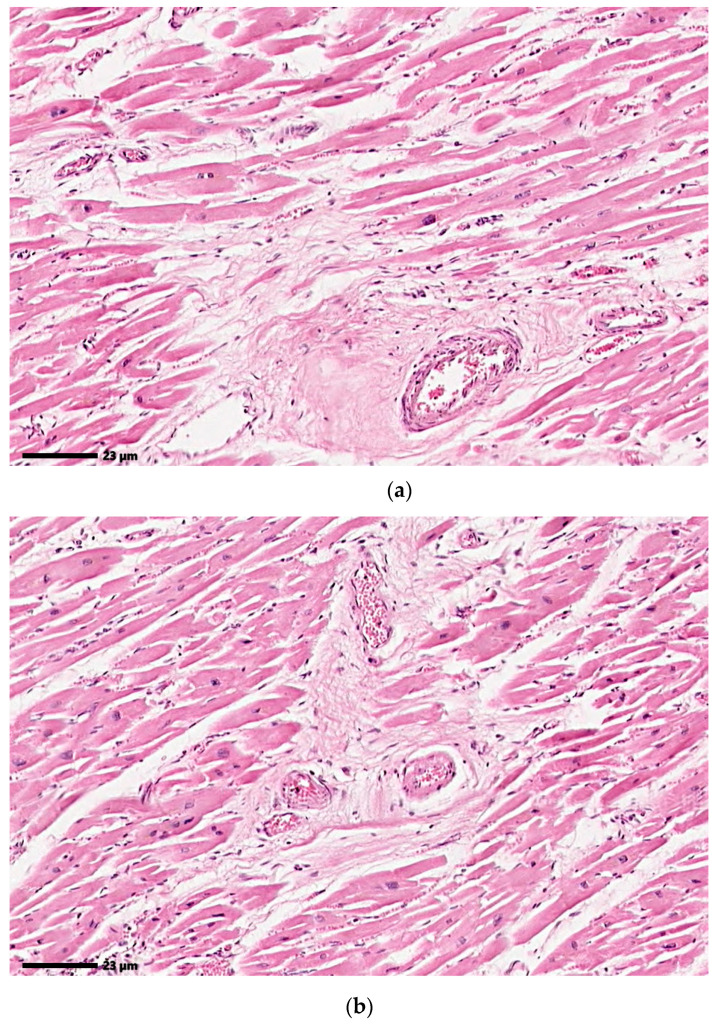
Histopathological images with small color variations between (**a**) fibrotic and (**b**) non-fibrotic areas. Original images: © The Iowa Virtual Slidebox [21].

However, in order to test the limitations of the system, the present research shows the use of 2 histopathological images in which the color difference between tissues with fibrosis and those without is not significant. The images analyzed are included in Figure 7.

Considering that the color variation in the histopathological images included in Figure 7 is not significant, which will lead to poor results for a method based on color analysis, the present research tested the Gabor filters for texture analysis.

**Figure 8:**
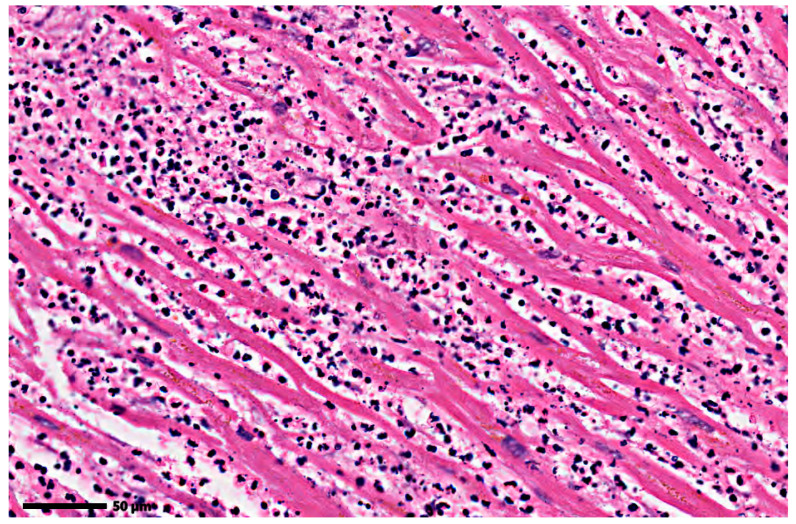
Myocardium without apparent fibrosis (recent myocardial infarction). Original image: © The Iowa Virtual Slidebox [21].

**Figure 9:**
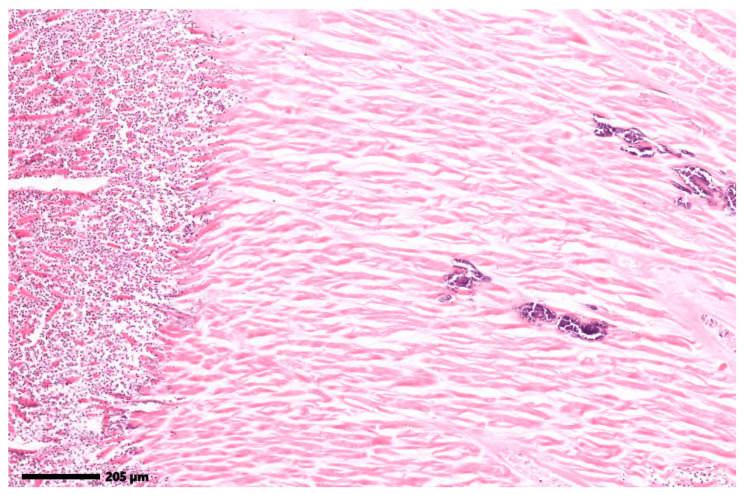
Myocardium without apparent fibrosis (septic infarct). Original image: © The Iowa Virtual Slidebox [21].

**Figure 10:**
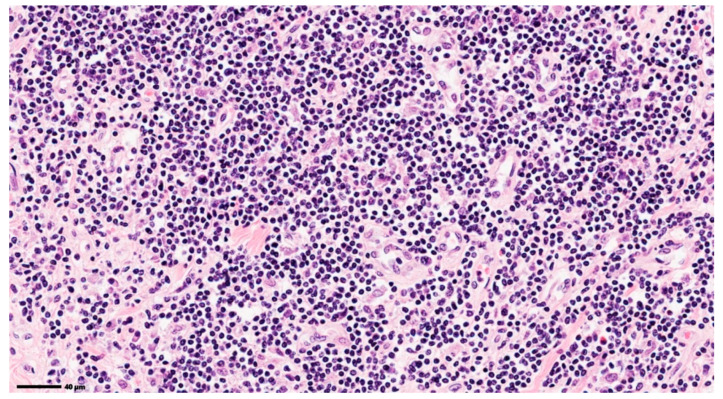
Histopathological image with heart transplant rejection. Original image: © The Iowa Virtual Slidebox [21].

In addition, for the same reason of evaluating the system’s accuracy, the obtained results were also analyzed in cases where potential confounders may be identified, due to a similar tinctorial affinity with the collagen deposition or to a fibrillar aspect. For an untrained person, these images can be interpreted as having fibrous tissue. As a result, the CIELAB spectrum-based analysis system was also examined under these conditions, on images with acute myocardial infarction (Figure 8), septic heart attack (Figure 9), and transplant rejection (Figure 10).

**Figure 11:**
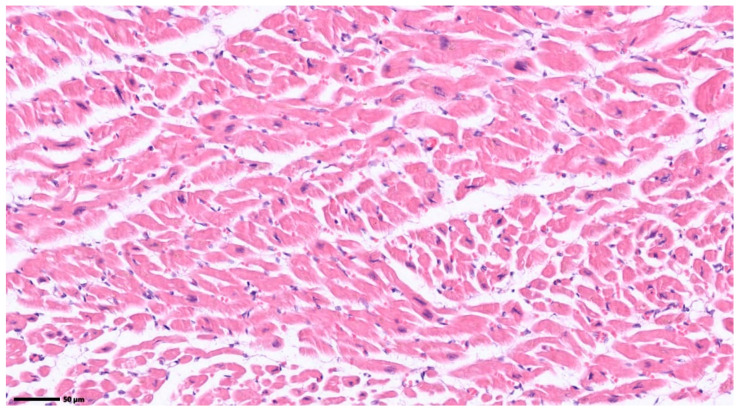

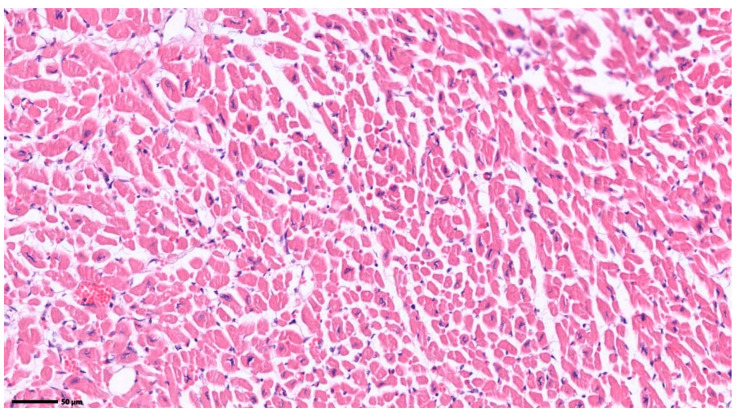
Histopathological images of normal tissue. Original image: © The Iowa Virtual Slidebox [21].

Moreover, given the semi-automated approach of selecting a region with fibrosis identified by the pathologist, the solution proposed in the present research was also tested on two histopathological images with normal tissue (images included in Figure 11).

### 2.3. Fibrosis Detection Using Gabor Filters

In order to increase the accuracy of the system presented in Section 2.2, the present research used a solution based on Gabor filters, where the previously presented solution shows its limitations. In this sense, Gabor filters replicate the way people distinguish textures and are used in programs that have this objective.

**Figure 12:**
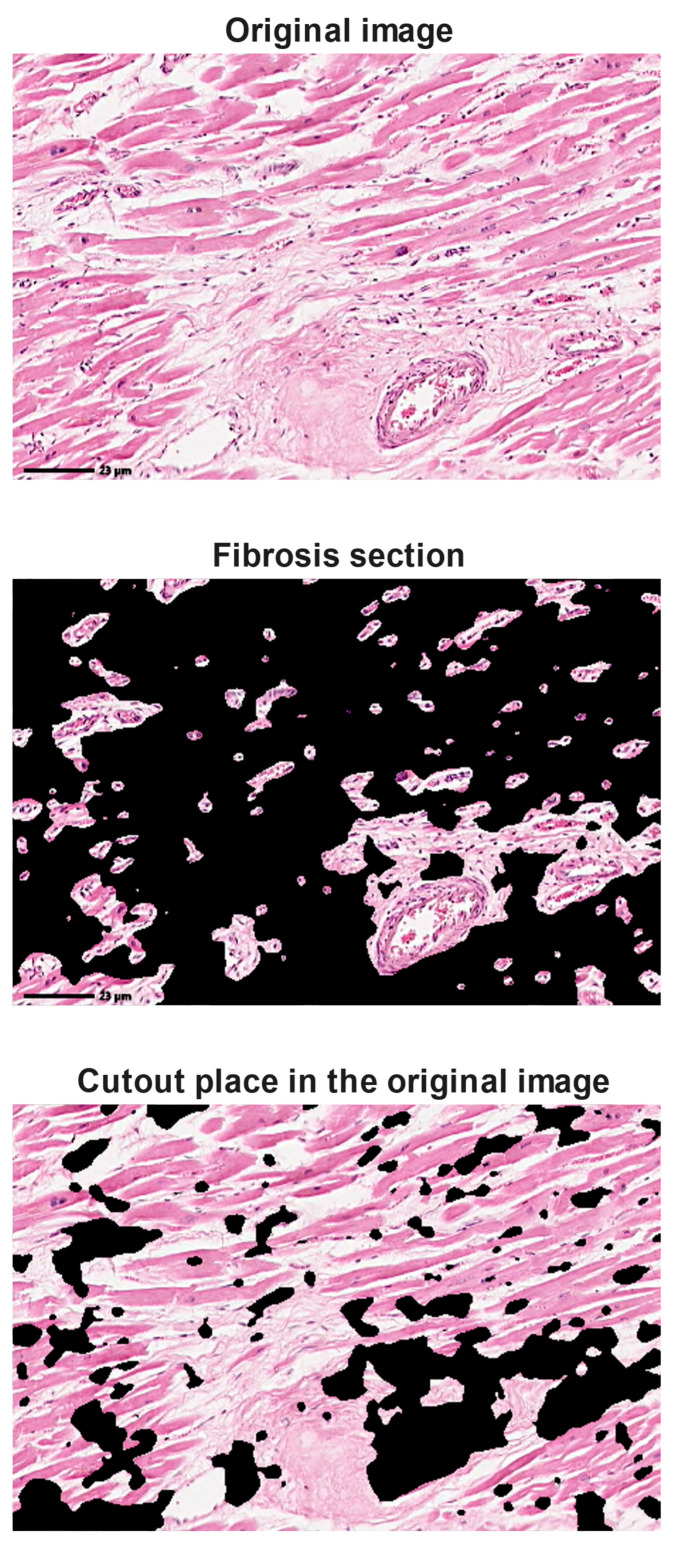
Graphical user interface of the results for MATLAB^®^ implementation of Gabor filters. Original image: © The Iowa Virtual Slidebox [21].

The system developed in the present research allows the user to choose an image. Then, automatically, three images are represented: the original image, the cropped region of the original image in which the tissue with fibrosis or with a difference in texture from the rest of the image, and the healthy tissue. To achieve this result, the automatic calculation process includes the definition of Gabor filters configured according to the image chosen by the user. Then, a Gaussian transformation is applied to smooth the image. Using the k-means information clustering method [31], the region with fibrosis or different tissue from the rest of the image is defined. The graphical user interface of the Gabor filters implementation is presented in Figure 12.

## 3. Results

### 3.1. Fibrosis Detection Using CIELAB Color Space

Since the use of CIELAB color space showed a higher accuracy of fibrosis identification from a visual point of view, a system was developed to evaluate the accuracy of the system using a source code developed in MATLAB^®^.

The accuracy evaluation program identifies the number of colored pixels different from the standard color (black for the automatic system and yellow for manual identification). In this respect, because the image sizes are different, the number of colored pixels was related to the image size, and a percentage was calculated. The results in terms of the number of pixels are included in Table 1 and Table 2. Table 3 contains the percentage comparison of pixels identified by the three uses: manual identification, and automatic identification with constant DeltaE tolerance for MATLAB^®^ and Python implementations.

Since no fibrosis could be identified manually at the pixel level, given the program for pixel-level accuracy analysis, it was considered a correct identification of the region with fibrosis if the percentage difference between the 2 cases was less than 10 units. This also highlights the main advantage of the automated fibrosis detection method, which is able to work at the pixel level, something that cannot be done manually by the pathologist. The last 5 images represent special categories: 41—acute myocardial infarction; 42—septic heart attack; 43—heart transplant rejection; 44 and 45—normal tissue.

As a result of the analysis of the results in Table 3, it appears that if the pathologist appropriately selects a small section of the region with fibrosis, the CIELAB color space-based method presented in this paper succeeds in correctly identifying the region with fibrosis in the histopathological images for cases where there is significant color variation.

In this case, out of 40 histopathological images with fibrosis, the MATLAB^®^ implementation of CIELAB color space analysis correctly identified fibrosis in 26 images. The Python implementation was successful in the identification of just seven images. However, the images in which fibrosis has been correctly identified differ between the two programming languages because, in the first situation, the user can choose a region with any geometric shape, while in Python, the selected region always has a rectangular shape. For the three special cases, both methods correctly identified a septic heart attack, representing one out of five special cases analyzed. The photomicrograph of transplant rejection and the relatively high percentage of collagen identified by the program illustrate the need for the judicious assessment of each case by the user, and highlight that the special stains are unsubstitutable. Indeed, there may be some collagen fibers, but they can be accurately identified only on special stains since other confounders have to be excluded (fibrin, hyaline material, amyloid, edema liquid, and cellular debris). If the pathologist does not select any region with fibrosis, the system does not detect fibrosis in the case of healthy tissue, resulting in the correct identification of tissue without fibrosis for the last 2 images tested. In this way, the CIELAB color space implementation in Python and MATLAB^®^ ensures a correct identification in four out of the five special situations analyzed. These images were processed correctly because the special tissue had a small weight in the histopathological image shown in Figure 9.

**Figure 13:**
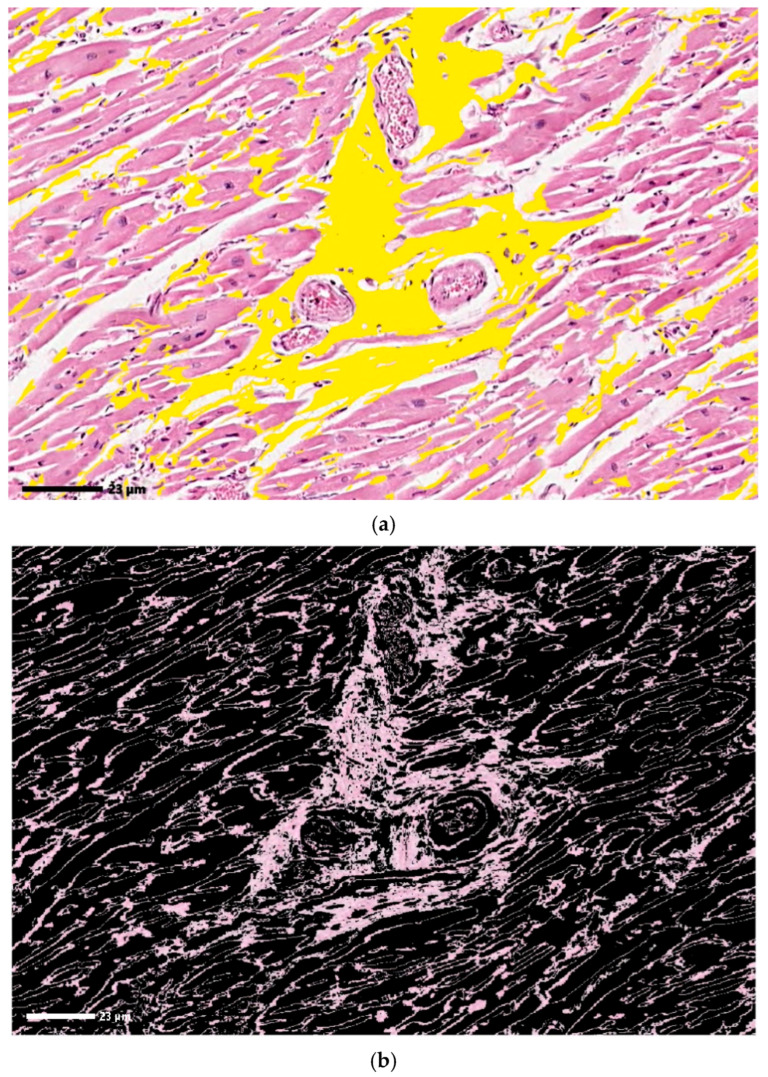
Manually (**a**) and automatically (**b**) identified fibrosis using MATLAB^®^ implementation of CIELAB color space-based system. Original image: © The Iowa Virtual Slidebox [21].

Figure 13 contains the color representation of fibrosis in histopathological image 11 in the case of manual (Figure 13a) and automated identification using CIELAB color space analysis (Figure 13b) implementation in MATLAB. As a result of the graphical interpretation of the images, it appears that the percentage analysis of tissue with fibrosis in the final image is an appropriate metric, and the system succeeds in adequately identifying tissue with fibrosis.

### 3.2. Fibrosis Detection Using Gabor Filters

As a result of Section 3.1, the present research shows the limitations of the CIELAB color space-based system: it does not return appropriate values in cases where the image contains fibrosis with colors similar to healthy tissue, or if the area examined is questionable and ancillary studies are needed.

Similarly to the tables in Section 3.1, Table 4 includes the size of the analyzed images for Gabor filter-based processing. Table 5 contains the number of pixels identified as having fibrosis by the manual and Gabor filter-based procedures, and Table 6 shows the percentages of fibrous tissue in the 45 images processed. Table 6 shows the results on the accuracy of the method based on Gabor filters compared to manual identification. Due to the lack of texture, the main differentiator of tissues when using Gabor filters, fibrosis is correctly identified in only 11 images by this method. It was considered correct identification if the percentage of fibrosis in the image is within ± 10% between the manually and automatically processed images with Gabor filters. The range of variation was determined based on statistical tests applied to the entire dataset. Even though this is a relatively large interval, given the manual identification performed by the pathologist, this interval led to statistically relevant results.

In the case of the Gabor filter-based implementation, the fibrosis region is correctly identified in 11 out of 40 cases, and it fails to correctly identify any particular case because it is not based on different textures. However, the objective of using Gabor filters was achieved: this implementation showed remarkable results in histopathological images 5, 6, 9, and 10, where the color difference between fibrous and healthy tissue was not significant. In these cases, the system based on color analysis in the CIELAB spectrum failed to return correct results, but the method based on Gabor filters was successful.

The implementation using Gabor filters showed its limitations when abnormal tissue, without fibrosis, is generalized to the whole analyzed image.

For a quantitative analysis of the results, the average percentage of fibrosis in the 45 analyzed images was determined for the situation where a manual identification is performed, or based on Gabor filters or CIELAB color space implemented in MATLAB^®^ and Python. Results on the accuracy of the methods are included in Table 7 for tissue with fibrosis, and in Table 8 for special tissue.

Considering the results presented in Table 7, it is identified that the average percentage of fibrosis is 30.37% in the case of manual identification for the 40 images containing fibrosis. The closest to this percentage was achieved using MATLAB^®^ implementation, with 22.37% fibrosis in the total image, and the Python implementation of CIELAB color space, with 38.13% in the analyzed images. Due to the difference in interpretation, based on textures, the Gabor filters identify the highest percentage of fibrosis in the analyzed images, leading to an average value of 54.97% for the average percentage of fibrosis identified by the Gabor method. Using a holistic approach, the three procedures achieved an accuracy of 38.49% of fibrosis, with an accuracy of 73.28%.

The accuracy of the procedures in terms of the percentage of fibrosis identified in images without fibrosis, included in Table 8, is kept the same as for those with fibrosis. Thus, the most accurate is the CIELAB method implemented in MATLAB^®^, with a percentage of 19.54% fibrosis, followed by the Python implementation with 36.79% of the same method and the Gabor filters with a value of 54.93%. These results lead to an accuracy of 62.91% for the combined methods when tested on images without fibrosis.

Table 9 contains an analysis of the overall accuracy of the semi-automated solution proposed in the present paper, which involves the integration of the three implementations for the identification of fibrosis in the 45 histopathological images analyzed.

Table 10 contains the number of errors for each of the three implementations for images with fibrosis and for the situation where the three methods are combined. The final accuracy of the system, 87.5%, is due to the fact that the system correctly identified the region with fibrosis in 35 of the 40 images with fibrosis. Table 11 contains the same analysis as in Table 10, but for the case of images with special tissue. In this case, out of the five images, only one was correctly identified as not containing fibrosis. However, the system relies on the pathologist’s input to select the region with fibrosis, so a specialist would not have selected anything in these last five images tested. The authors of this study chose a tissue similar to fibrosis in order to evaluate the influence that the pathologist’s experience has on the accuracy of the diagnostic system. Based on the results obtained, the system has demonstrated that it correlates with the pathologist’s expertise and cannot be used in the learning process, but it can be extremely useful for specialists to facilitate their work after they have acquired the necessary experience. Table 12 represents the accuracy of using the 3 implementations for all 45 histopathological images. In this regard, the system returned valid responses for 36 of the 45 cases tested.

## 4. Discussion

Following the obtained results, the solution developed in the present research regarding the identification of fibrosis in histopathological images comprises a holistic approach, based on the use of CIELAB color space and Gabor filters. In the case of the implementation presented in this paper, the pathologist is responsible for choosing a small area with fibrosis from a slide. Employing this semi-automated approach, the present research showed the advantages of each implementation and presented a semi-automated detection system for fibrosis in histopathological images using a holistic approach based on both CIELAB color space and Gabor filters. While manual identification required a qualitative and quantitative analysis of the entire image by the pathologist, the proposed semi-automatic detection system reduces the analysis time to the detection of a region, no matter how small, in the image that contains fibrosis. The system then automatically evaluates the amount of fibrosis in the entire image without requiring input from the pathologist. This reduces the analysis time for experienced pathologists, providing a quantitative assessment that can be used as a reference for future comparisons.

The authors, however, are aware of this study’s limitations. One major constraint of the research is determined by the pathologist’s expertise: the results are considerably altered if invalid regions are chosen. This shortcoming is decreased as the pathologist’s expertise increases. Another limitation is that the authors proposed the identification of fibrotic areas on hematoxylin and eosin-stained slides, instead of using a special stain for collagen. This might lead to errors in the assessment of fibrosis areas, taking into account the existence of other deposits in the extracellular space that can mimic fibrosis (amyloid, edema fluid, hyaline material). Moreover, cases with subtle collagen deposits can easily be missed in hematoxylin and eosin-stained slides. In this regard, the database was made of images with clear fibrotic areas and is suitable for use in a semi-automatic approach, aiding the pathologist in the evaluation and contributing, together with the human evaluation, to improved and accurate diagnosis. Furthermore, the limited number of images used in this study cannot be used as a basis for generally valid truths, and another important limitation is this study’s use of a single database of whole-slide images. In addition, the pixel-level overlap was validated using the pathologist’s expertise. As a result, it will be necessary in the future to increase the database size and analyze performance at the pixel level of a larger dataset using multiple objective performance metrics like dice, sensitivity, or specificity. In addition, the Gabor filters tend to significantly overestimate the fibrosis rate in many images, indicating that the approach is prone to over-segmentation. This likely occurs because the filter responds strongly to texture patterns that are not exclusively associated with fibrotic regions, causing non-fibrotic structures to be incorrectly classified as fibrosis. As a result, the detected fibrosis area is artificially inflated, suggesting that additional post-processing or more discriminative features may be necessary to improve segmentation accuracy. It should also be noted that fibrosis in this study was identified manually by a single pathologist. Considering the issue of inter-observer variability, future analyses should incorporate the assessments of at least two pathologists.

## 5. Conclusions

The present paper discusses the possibility of fibrosis detection using CIELAB color space and Gabor filters. Herein, a semi-automated system for the detection of collagenous areas on hematoxylin and eosin-stained slides is developed. The best-obtained accuracy using only CIELAB color space is 74.46% [24] on all 40 histopathological images with fibrotic tissue. Under identical conditions and using the same image dataset, the newly proposed system, which applies Gabor filters combined with the CIELAB color space, achieved an accuracy rate of 87.5%. However, when the images either lack fibrosis or have poor color contrast between normal and abnormal tissue, the system’s accuracy drops to 80% across the entire dataset of 45 histopathological images. Nevertheless, this percentage surpasses the 74.46% accuracy achieved when utilizing solely the CIELAB color space. In the present research, when implementing the combined method mentioned above on images featuring fibrotic tissue, the newly developed system achieves an accuracy of 87.50%, considering the 10% margin of difference between manual and automatic identification, given the impossibility of high-accuracy identification by the method of manual marking of fibrosis by the pathologist. As a result, due to the application of the method at the pixel level, the accuracy is higher than for manual identification, which is a real help for pathologists. This outcome demonstrates the effectiveness of the proposed approach.

Future research directions are focused on identifying other machine learning-based methods for fibrosis detection to further increase the accuracy of the method and on developing a solution that is not decisively influenced by user experience using machine learning-based methods. In addition, another research direction is the development of metrics to analyze the performance of fibrosis identification, not only based on the percentage area identified as having fibrosis but also through a comparative analysis of the regions identified by the pathologist and those automatically detected using the method proposed in this paper.

## Figures and Tables

**Table 1:** Size of the analyzed images using CIELAB color space.

Image Number	Original Image Size	Image Size Generated for MATLAB^®^ Implementation	Image Size Generated for Python Implementation
1	921 × 616	1155 × 774	921 × 616
2	921 × 616	1155 × 774	921 × 616
3	921 × 616	1155 × 774	921 × 616
4	921 × 616	1155 × 774	921 × 616
5	921 × 616	1155 × 774	921 × 616
6	921 × 616	1155 × 774	921 × 616
7	921 × 616	1155 × 774	921 × 616
8	921 × 616	1155 × 774	921 × 616
9	921 × 616	1155 × 774	921 × 616
10	921 × 616	1155 × 774	921 × 616
11	921 × 616	1155 × 774	921 × 616
12	1115 × 616	1398 × 774	1115 × 616
13	1115 × 616	1398 × 774	1115 × 616
14	1115 × 616	1398 × 774	1115 × 616
15	1115 × 616	1398 × 774	1115 × 616
16	1115 × 616	1398 × 774	1115 × 616
17	1115 × 616	1398 × 774	1115 × 616
18	937 × 616	1175 × 774	937 × 616
19	937 × 616	1175 × 774	937 × 616
20	937 × 616	1175 × 774	937 × 616
21	937 × 616	1175 × 774	937 × 616
22	937 × 616	1175 × 774	937 × 616
23	937 × 616	1175 × 774	937 × 616
24	937 × 616	1175 × 774	937 × 616
25	937 × 616	1175 × 774	937 × 616
26	937 × 616	1175 × 774	937 × 616
27	937 × 616	1175 × 774	937 × 616
28	937 × 616	1175 × 774	937 × 616
29	937 × 616	1175 × 774	937 × 616
30	937 × 616	1175 × 774	937 × 616
31	937 × 616	1175 × 774	937 × 616
32	937 × 616	1175 × 774	937 × 616
33	937 × 616	1175 × 774	937 × 616
34	937 × 616	1175 × 774	937 × 616
35	937 × 616	1175 × 774	937 × 616
36	937 × 616	1175 × 774	937 × 616
37	937 × 616	1175 × 774	937 × 616
38	937 × 616	1175 × 774	937 × 616
39	937 × 616	1175 × 774	937 × 616
40	937 × 616	1175 × 774	937 × 616
41	937 × 616	1175 × 774	937 × 616
42	937 × 616	1175 × 774	937 × 616
43	937 × 616	1175 × 774	937 × 616
44	937 × 616	1175 × 774	937 × 616
45	937 × 616	1175 × 774	937 × 616

**Table 2:** The number of pixels identified as having fibrous tissue using CIELAB color space.

Image Number	Number of Fibrosis Pixels for Manual Identification	Number of Fibrosis Pixels for MATLAB^®^ Implementation	Number of Fibrosis Pixels for Python Implementation
1	215,716	148,047	138,737
2	140,224	119,555	140,706
3	240,895	149,127	144,542
4	270,748	172,357	160,072
5	386,553	231,284	257,170
6	410,440	217,715	223,937
7	252,138	164,117	157,676
8	315,264	172,637	159,021
9	342,680	218,218	259,063
10	307,601	203,399	195,596
11	177,975	137,546	141,415
12	63,795	208,554	235,216
13	73,447	210,744	238,345
14	167,816	269,490	301,505
15	82,008	202,507	274,541
16	115,202	232,695	283,967
17	112,765	236,892	266,986
18	133,159	277,477	332,811
19	132,623	284,239	275,741
20	178,399	279,763	304,097
21	118,927	231,046	260,216
22	35,206	108,527	120,321
23	145,561	168,318	232,954
24	171,292	320,456	280,337
25	179,593	211,797	291,002
26	334,536	358,449	325,038
27	95,262	201,323	263,779
28	109,211	180,626	222,184
29	79,511	174,023	177,446
30	111,606	205,574	213,678
31	100,889	158,361	181,136
32	315,414	314,985	285,941
33	70,368	153,468	171,881
34	248,720	185,230	215,320
35	162,835	174,455	217,815
36	139,449	273,355	224,637
37	113,001	206,461	218,245
38	57,819	148,589	167,735
39	182,551	231,942	271,317
40	167,528	178,021	194,018
41	0	176,801	158,203
42	0	186,446	109,634
43	0	16,737	40,011
44	0	107,014	365,001
45	0	401,827	389,076

**Table 3:** Accuracy of the CIELAB color space-based system using MATLAB^®^ and Python implementations.

Image Number	Fibrosis Percentage in the Image: Manual Identification [%]	Fibrosis Percentage in the Image: MATLAB^®^ Implementation [%]	Fibrosis Percentage in the Image: Python Implementation [%]
1	38.02	16.56	24.45
2	24.72	13.37	24.80
3	42.46	16.68	25.48
4	47.72	19.28	28.21
5	68.13	25.87	45.33
6	72.35	24.35	39.47
7	44.44	18.36	27.79
8	55.57	19.31	28.03
9	60.40	24.41	45.66
10	54.22	22.75	34.48
11	31.37	15.39	24.93
12	9.29	19.27	34.25
13	10.69	19.48	34.70
14	24.43	24.91	43.90
15	11.94	18.72	39.97
16	16.77	21.50	41.34
17	16.42	21.89	38.87
18	23.07	30.51	57.66
19	22.98	31.25	47.77
20	30.91	30.76	52.69
21	20.60	25.41	45.08
22	6.10	11.93	20.85
23	25.22	18.51	40.36
24	29.68	35.24	48.57
25	31.11	23.29	50.42
26	57.96	39.41	56.31
27	16.50	22.14	45.70
28	18.92	19.86	38.49
29	13.78	19.13	30.74
30	19.34	22.60	37.02
31	17.48	17.41	31.38
32	54.65	34.63	49.54
33	12.19	16.87	29.78
34	43.09	20.37	37.30
35	28.21	19.18	37.74
36	24.16	30.06	38.92
37	19.58	22.70	37.81
38	10.02	16.34	29.06
39	31.63	25.50	47.01
40	29.02	19.57	33.61
41	0	19.44	27.41
42	0	20.50	18.99
43	0	1.84	6.93
44	0	11.77	63.24
45	0	44.18	67.41

**Table 4:** Size of the analyzed images using Gabor filters.

Image Number	Original Image Size	Image Size Generated for Gabor Filters Implementation
1	921 × 616	1155 × 774
2	921 × 616	1155 × 774
3	921 × 616	1155 × 774
4	921 × 616	1155 × 774
5	921 × 616	1155 × 774
6	921 × 616	1155 × 774
7	921 × 616	1155 × 774
8	921 × 616	1155 × 774
9	921 × 616	1155 × 774
10	921 × 616	1155 × 774
11	921 × 616	1155 × 774
12	1115 × 616	1398 × 774
13	1115 × 616	1398 × 774
14	1115 × 616	1398 × 774
15	1115 × 616	1398 × 774
16	1115 × 616	1398 × 774
17	1115 × 616	1398 × 774
18	937 × 616	1175 × 774
19	937 × 616	1175 × 774
20	937 × 616	1175 × 774
21	937 × 616	1175 × 774
22	937 × 616	1175 × 774
23	937 × 616	1175 × 774
24	937 × 616	1175 × 774
25	937 × 616	1175 × 774
26	937 × 616	1175 × 774
27	937 × 616	1175 × 774
28	937 × 616	1175 × 774
29	937 × 616	1175 × 774
30	937 × 616	1175 × 774
31	937 × 616	1175 × 774
32	937 × 616	1175 × 774
33	937 × 616	1175 × 774
34	937 × 616	1175 × 774
35	937 × 616	1175 × 774
36	937 × 616	1175 × 774
37	937 × 616	1175 × 774
38	937 × 616	1175 × 774
39	937 × 616	1175 × 774
40	937 × 616	1175 × 774
41	937 × 616	1175 × 774
42	937 × 616	1175 × 774
43	937 × 616	1175 × 774
44	937 × 616	1175 × 774
45	937 × 616	1175 × 774

**Table 5:** Number of pixels identified as having fibrous tissue using Gabor filters.

Image Number	Number of Fibrosis Pixels for Manual Identification	Number of Fibrosis Pixels for Gabor Filters Implementation
1	215,716	541,410
2	140,224	326,481
3	240,895	570,221
4	270,748	739,971
5	386,553	634,222
6	410,440	663,299
7	252,138	591,254
8	315,264	353,707
9	342,680	516,978
10	307,601	437,666
11	177,975	328,988
12	63,795	399,743
13	73,447	395,091
14	167,816	741,204
15	82,008	485,990
16	115,202	547,571
17	112,765	811,449
18	133,159	624,545
19	132,623	592,188
20	178,399	212,797
21	118,927	554,155
22	35,206	463,475
23	145,561	516,142
24	171,292	457,849
25	179,593	807,038
26	334,536	584,035
27	95,262	747,961
28	109,211	244,845
29	79,511	638,162
30	111,606	642,990
31	100,889	247,407
32	315,414	490,152
33	70,368	514,503
34	248,720	562,219
35	162,835	471,774
36	139,449	460,182
37	113,001	359,327
38	57,819	407,001
39	182,551	358,105
40	167,528	396,547
41	0	407,651
42	0	393,731
43	0	663,970
44	0	661,809
45	0	370,744

**Table 6:** Accuracy of the Gabor filter-based system.

Image Number	Fibrosis Percentage in the Image: Manual Identification [%]	Fibrosis Percentage in the Image: Gabor Filters Implementation [%]
1	38.02	60.56
2	24.72	36.52
3	42.46	63.79
4	47.72	82.77
5	68.13	70.94
6	72.35	74.20
7	44.44	66.14
8	55.57	39.57
9	60.40	57.83
10	54.22	48.96
11	31.37	36.80
12	9.29	36.94
13	10.69	36.51
14	24.43	68.50
15	11.94	44.91
16	16.77	50.60
17	16.42	74.99
18	23.07	68.67
19	22.98	65.11
20	30.91	23.40
21	20.60	60.93
22	6.10	50.96
23	25.22	56.75
24	29.68	50.34
25	31.11	88.74
26	57.96	64.22
27	16.50	82.24
28	18.92	26.92
29	13.78	70.17
30	19.34	70.70
31	17.48	27.20
32	54.65	53.90
33	12.19	56.57
34	43.09	61.82
35	28.21	51.87
36	24.16	50.60
37	19.58	39.51
38	10.02	44.75
39	31.63	39.38
40	29.02	43.60
41	0	44.82
42	0	43.29
43	0	73.01
44	0	72.77
45	0	40.77

**Table 7:** Accuracy of methods tested using images with fibrosis.

Type of Identification Method	Average Percentage of Fibrosis [%]	Accuracy [%]
Manual identification	30.3786	100.00
CIELAB (Python)	38.1372	74.4602
CIELAB (MATLAB^®^)	22.3701	73.6376
Gabor filters	54.9730	19.04
Proposed system	38.4934	73.2875

**Table 8:** Accuracy of methods tested using images with special tissue.

Type of Identification Method	Average Percentage of Fibrosis [%]	Accuracy [%]
Manual identification	0	100.00
CIELAB (Python)	36.7962	63.2038
CIELAB (MATLAB^®^)	19.5464	80.4534
Gabor filters	54.9322	45.0678
Proposed system	37.0916	62.9084

**Table 9:** Correct identification of fibrosis.

Image Number	CIELAB (MATLAB^®^) Implementation	CIELAB (Python) Implementation	Gabor Filters Implementation
1			
2	YES		
3			
4			
5			YES
6			YES
7			
8			
9			YES
10			YES
11	YES		YES
12		YES	
13		YES	
14		YES	
15		YES	
16		YES	
17		YES	
18		YES	
19		YES	
20		YES	YES
21		YES	
22		YES	
23		YES	
24		YES	
25		YES	
26	YES		YES
27		YES	
28		YES	YES
29		YES	
30		YES	
31		YES	YES
32	YES		YES
33		YES	
34	YES		
35	YES	YES	
36		YES	
37		YES	
38		YES	
39		YES	YES
40	YES	YES	
41			
42			
43	YES	YES	
44			
45			

**Table 10:** Combined accuracy of the three implementations for fibrosis.

Type of Identification Method	Errors	Accuracy [%]
Manual identification	0	100.00
CIELAB (Python)	33	17.5
CIELAB (MATLAB^®^)	14	65
Gabor filters	29	27.5
Proposed system	5	87.5

**Table 11:** Combined accuracy of the three implementations for special tissue.

Type of Identification Method	Errors	Accuracy [%]
Manual identification	0	100.00
CIELAB (Python)	4	20
CIELAB (MATLAB^®^)	4	20
Gabor filters	5	0
Proposed system	4	20

**Table 12:** Combined accuracy of the three implementations for all images.

Type of Identification Method	Errors	Accuracy [%]
Manual identification	0	100.00
CIELAB (Python)	37	17.77
CIELAB (MATLAB^®^)	18	60
Gabor filters	34	24.44
Proposed system	9	80

## Data Availability

The data presented in this study are openly available in Iowa Virtual Slidebox at https://www.biolucida.net/images/?page=images (accessed on 20 July 2024), reference number [21].
